# Accelerated epigenetic aging and myopenia in young adult cancer survivors

**DOI:** 10.1002/cam4.5908

**Published:** 2023-04-09

**Authors:** Stephanie C. Gehle, Daniel Kleissler, Hillary Heiling, Allison Deal, Zongli Xu, Vanessa L. Ayer Miller, Jack A. Taylor, Andrew B. Smitherman

**Affiliations:** ^1^ Department of Pediatrics UNC School of Medicine University of North Carolina at Chapel Hill Chapel Hill North Carolina USA; ^2^ Lineberger Comprehensive Cancer Center University of North Carolina at Chapel Hill Chapel Hill North Carolina USA; ^3^ Department of Biostatistics at the Gillings School of Global Public Health University of North Carolina at Chapel Hill Chapel Hill North Carolina USA; ^4^ Epidemiology Branch National Institute of Environmental Health Sciences Durham North Carolina USA; ^5^ College of Pharmacy and Health Sciences Campbell University Buies Creek North Carolina USA

**Keywords:** aging, cancer survivorship, DNA methylation, epigenetic age

## Abstract

**Background:**

Young adult cancer survivors experience early aging‐related morbidities and mortality. Biological aging biomarkers may identify at‐risk survivors and increase our understanding of mechanisms underlying this accelerated aging.

**Methods:**

Using an observational study design, we cross‐sectionally measured DNA methylation‐based epigenetic age in young adult cancer survivors at a tertiary, academic state cancer hospital. Participants were a convenience sample of consecutively enrolled survivors of childhood, adolescent, and young adult cancers treated with either an anthracycline or alkylating agent, and who were at least 3 months post‐treatment. Similarly aged healthy comparators were consecutively enrolled. Cancer treatment and treatment intensity were compared to DNA methylation‐based epigenetic age and pace of aging.

**Results:**

Sixty survivors (58 completing assessments, mean age 20.5 years, range 18–29) and 27 comparators (mean age 20 years, range 17–29) underwent DNA methylation measurement. Survivors were predominantly female (62%) and white (60%) and averaged nearly 6 years post‐treatment (range 0.2–25 years). Both epigenetic age (AgeAccelGrim: 1.5 vs. −2.4, *p* < 0.0001; AgeAccelPheno 2.3 vs. −3.8, *p* = 0.0013) and pace of aging (DunedinPACE 0.99 vs. 0.83, *p* < 0.0001) were greater in survivors versus comparators. In case–case adjusted analysis, compared to survivors with normal muscle mass, myopenic survivors had higher AgeAccelGrim (2.2 years, 95% CI 0.02–4.33, *p* = 0.02), AgeAccelPheno (6.2 years, 2.36–10.09, *p* < 0.001), and DunedinPACE (0.11, 0.05–0.17, *p* < 0.001).

**Conclusions:**

Epigenetic age is older and pace of aging is faster in young adult cancer survivors compared to noncancer peers, which is evident in the early post‐therapy period. Survivors with physiological impairment demonstrate greater epigenetic age advancement. Measures of epigenetic age may identify young adult survivors at higher risk for poor functional and health outcomes.

## Graphical abstract

In this cohort study of 58 young adult cancer survivors and 27 comparably aged, noncancer comparators, epigenetic age was greater among survivors compared to controls. Among survivors, greater epigenetic age was associated with myopenia and frailty. Young adult cancer survivors have greater epigenetic age potentially providing a useful tool to assess risk for physiological impairment, disability, and poor health.

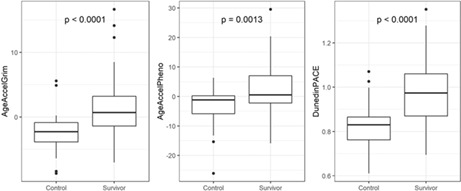

## 1 INTRODUCTION

Advancements in cancer and supportive therapies have led to a growing population of young adult survivors^1^, ^2^ with more than 700,000 survivors now living in the United States.^3^ The majority of young adult survivors experience significant impairments in health, quality of life, and lifespan with increased secondary cancers, cardiovascular disease, and endocrinopathies.^4^, ^5^, ^6^, ^7^, ^8^, ^9^, ^10^, ^11^, ^12^, ^13^, ^14^, ^15^ The premature onset of aging‐related morbidities and functional impairment point to a process of accelerated aging in survivors, the underlying mechanism of which is not well understood.^7^, ^16^, ^17^ Biomarkers of biological aging, developed from knowledge of the hallmarks of human aging,^18^, ^19^, ^20^, ^21^ may identify accelerated aging before the onset of functional declines and morbidities and characterize populations at higher risk for aging‐related adverse outcomes.^7^, ^16^, ^22^, ^23^ Additionally, measuring aging biomarkers in survivors may lead to a better understanding of the mechanisms underlying accelerated aging in this population, guide development of interventions to halt or mitigate this process, and serve as more proximate outcomes for interventional studies.^16^, ^23^, ^24^

Measures of epigenetic age using DNA methylation‐based “clocks” provide an easily assessable and reliable method for characterizing biological age.^25^, ^26^, ^27^, ^28^, ^29^, ^30^, ^31^, ^32^ These clocks use predictable patterns of DNA methylation across the epigenome to estimate an individual's age, and when adjusted for chronological age, may identify accelerated aging. Later‐generation epigenetic clocks not only identify accelerated aging but also predict risk for the development of morbidities such as cardiovascular disease and early mortality.^28^, ^31^, ^33^, ^34^, ^35^, ^36^, ^37^ The PhenoAge epigenetic clock was designed to estimate a previously developed “phenotypic age score”—a score based on chronological age and nine blood measures (albumin, creatinine, glucose, C‐reactive protein, alkaline phosphatase, white blood cell count, lymphocyte percent, red blood cell width and volume) that is correlated with age‐related disease.^34^ The GrimAge epigenetic clock was developed by identifying patterns of methylation that could be used as DNA methylation‐based estimators of factors associated with aging and morbidity—seven plasma proteins (adrenomedullin, beta‐2‐microglobulin, cystatin C, growth differentiation factor 15, leptin, PAI‐1, and tissue inhibitor metalloproteinase‐1) and smoking history (pack years). GrimAge incorporates these DNAm estimators along with chronological age and sex.^37^ For both PhenoAge and GrimAge the difference (calculated using residuals from linear models regressing biological age on chronological age) between a person's DNAm biological age and their known chronological age is termed “Age Acceleration” (PhenoAgeAccel and GrimAgeAccel, respectively). Although the two underlying clocks are highly correlated with age, by design both of the age acceleration metrics are independent of chronological age. A third measure DunedinPACE, uses DNAm to estimate a previously developed “Pace of Aging” metric. The original Pace of Aging metric was based on changes in nineteen biomarkers of cardiovascular, metabolic, renal, hepatic, immune, dental, and pulmonary systems that were serially measured in a birth cohort over the course of 20 years.^35^ Unlike the two age acceleration metrics, DunedinPACE has a mean value of 1—reflecting an average year of age‐related change observed in the original cohort study. This pace of aging measure may be thought of as estimate of aging rate, and unlike the age acceleration metrics, increases slightly with increasing chronological age.

Accelerated epigenetic age has been observed in long‐term survivors of childhood cancers and was associated with higher risks for chronic morbidities.^38^ Epigenome‐wide association studies have identified treatment‐associated DNA methylation changes that persist decades after therapy. Some of these epigenetic changes were associated with the development of cardiovascular risk factors suggesting these epigenetic alterations may, in part, mediate the link between treatment exposures and risk for adverse late effects.^39^

Using an observational study design, we cross‐sectionally measured epigenetic age and pace of aging in young adult survivors of childhood, adolescent, and young adult cancers hypothesizing that survivors would have older epigenetic age and faster pace of aging versus comparators and that epigenetic aging would be even greater among survivors with physiological evidence of aging (myopenia or frailty).

## 2 METHODS

### 2.1 Study population

A convenience sample of consecutive survivors of childhood, adolescent, or young adult cancers were enrolled through the University of North Carolina (UNC) Children's Oncology Clinic or the UNC Adolescent and Young Adult Survivors' Clinic. Eligible survivors were treated with an anthracycline, an anthracenedione, and/or an alkylating agent and were returning for a routine survivorship appointment. Patients who received stem cell transplants were excluded. Cancer‐free comparators of a similar age range were consecutively enrolled by convenience sampling from either the UNC Children's Hematology Clinic (seen for evaluation of benign conditions such as iron‐deficiency anemia) or the UNC Platelet Donation Center. After informed consent, a peripheral blood sample was drawn in conjunction with routine clinical care and survivors were asked to complete self‐report measures and undergo physical function assessments.

All study activities were approved by both the UNC Lineberger Comprehensive Cancer Center Protocol Review Committee and the UNC IRB.

### 2.2 Measures and outcomes

Survivors completed self‐report assessments of health, quality of life, and physical function (the Medical Outcomes Study 36‐item Short‐Form Health Survey^40^ and the National Health and Nutrition Examination Survey physical activity questionnaire^41^), were assessed by study team members for 13 CTCAE‐graded common conditions and symptoms, underwent body composition analysis for measurement of total body skeletal muscle, completed a 15‐foot timed walk, and underwent grip strength measurement.

Bioelectrical impedance analysis (BIA) was used to measure total body skeletal muscle mass on a Quantum IV body composition analyzer (RJL Systems). Resistance (ohm) was measured at an operating frequency of 50 kHz at 800 μA. Skeletal muscle mass (kg) was derived from BIA impedance values according to the equation previously reported by Janssen also using an RJL Systems body composition analyzer.^42^ Skeletal muscle index (SMI) was derived by dividing the skeletal muscle mass by the total mass (kg). *Myopenia* was defined as an SMI ≤0.37 for men and ≤0.28 for women, which correlates to one standard deviation below population means among young adults.^43^, ^44^

We defined frailty using the phenotype developed by Fried et al.^45^ and modified by Ness.^46^, ^47^ The frailty phenotype is measured using five factors: myopenia, weakness, slowness, exhaustion, and low energy expenditure. Frailty was defined as having ≥3 factors, prefrailty as having 2 factors, and nonfrail status as having ≤1 factor. *Myopenia* was defined as above. *Weakness* was measured in the dominant hand with a hand‐held hydraulic dynamometer (model J00105; Lafayette Instrument while in a seated position with the forearm flexed at the elbow to 90°).^45^ Weakness was classified using the mean of two measures and cutoffs reported by Fried based on sex and body mass index.^45^ *Slowness* was measured with a timed 15‐foot walk at the survivor's usual pace on a hard, noninclined surface. Women ≤159 cm and men ≤173 cm in height were considered slow with a time ≥7 s. Women >159 cm and men >173 cm in height were considered slow with a time ≥6 s.^45^ No accommodations were made for individuals who had health concerns affecting gait, such as neuropathy, ataxia, or prosthesis. *Exhaustion* was determined using the Medical Outcomes Study 36‐item Short‐Form Health Survey vitality subscale.^40^ A subscale score ≤ 40 defined exhaustion, which correlates to one standard deviation below the population mean. Leisure time *energy expenditure* was measured using the National Health and Nutrition Examination Survey physical activity questionnaire.^45^ Low energy expenditure was defined as <383 kcal/week for men and <270 kcal/week for women.^45^

Members of the study team completed standardized abstraction of medical records for each survivor to collect details on demographics, cancer, chronic medical conditions, and cancer treatment. Total doses were calculated for anthracyclines/anthracenediones in doxorubicin equivalents^48^ and for alkylators in cyclophosphamide equivalents.^49^ Comorbidities were determined from medical record abstraction. Common cancer‐related symptoms were collected using a standardized form of 13 CTCAE‐graded conditions administered by study team members. The intensity of treatment was standardized using a validated measure, the Intensity of Treatment Rating Scale, version 3 (ITR‐3).^50^ Regimens were classified according to the ITR‐3 score from lowest (1) to highest (4) intensity. A pediatric and an AYA oncologist who were blinded to patient outcomes independently assigned intensity scores with 100% agreement.

### 2.3 Epigenetic clock analysis

Using whole blood collected in EDTA tubes, extraction and isolation of DNA was performed by the UNC Biospecimen Processing Facility. DNA was isolated using the IonicR Cells to Pure DNA Kit (Purigen Biosystems, 33005). Bisulfite conversion was performed using the Zymo EZ DNA Methylation‐Gold Kit (Zymo, D5006). Up to 750 ng of DNA was converted using the standard manufacturer protocol. DNA methylation analysis was performed by the Mammalian Genotyping Core at the Lineberger Comprehensive Care Center using combined manual and automatic (robotic) protocols for the Illumina Infinium MethylationEPIC Assay per standard protocol.^28^ Methylation profiling of 865,918 CpGs were performed using Illumina Infinium HumanMethylationEPIC BeadChip. The raw DNA methylation data were preprocessed to improve data quality with the following steps: reducing background noise with ENmix method^51^; correcting fluorescent dye‐bias using RELIC method^52^; quantile normalization to ensure overall DNA methylation intensity distribution comparable across samples, and reducing Infinium I and II probe design bias using RCP method.^53^ One sample was excluded because the percentage of low‐quality CpG values (detection *p* > 0.000001 or number of beads <3) was greater than 0.02. A total of 18,300 CpGs with a percentage of low‐quality values greater than 0.05 were also excluded. The normalized DNA methylation beta values for a subset of the CpGs were utilized to calculate validated DNA methylation clocks using the publicly available DNA methylation age calculators. Estimated epigenetic age was calculated using PhenoAge^34^ (513 CpGs), and GrimAge (1030 CpGs)^37^ clocks. AgeAccelPheno and AgeAccelGrim, measures of epigenetic age acceleration, are derived using residuals from the regression of epigenetic age and chronological age. The DunedinPACE^35^ (Pace of Aging Calculated from the Epigenome) measure was also calculated to measure each participant's ongoing rate of aging based on decline in organ systems function over time. DunedinPACE estimates aging rate, and unlike the age acceleration metrics, increases slightly with increasing chronological age.

### 2.4 Statistical analysis

Patient characteristics were summarized using descriptive statistics, including median and range for continuous measures and frequencies for categorical measures. Tests for differences in the aging acceleration measures (AgeAccelGrim, AgeAccelPheno) and the pace of aging measure (DunedinPACE) between the survivor and comparator groups were conducted using two‐sample *t*‐tests. Associations between the three biological aging outcomes and survivor characteristics were assessed using linear regression models. Multivariable linear regression models were used to assess the association between the aging outcomes in the survivors and frailty and myopenia while adjusting for sex, race, ITR‐3 treatment intensity levels, and time off treatment. Sex, race, ITR‐3 levels, and time off treatment were adjusted for in the models as they were statistically significant (*p* < 0.05) with at least one of the three aging outcomes in the bivariable association analyses. Differences in SF‐36 scores between survivors with and without age acceleration (AgeAccelPheno or Age AccelGrim > 0) or with and without accelerated pace of aging (DunedinPACE > 1) were tested using two‐sample *t*‐tests.

## 3 RESULTS

DNA methylation and epigenetic age were measured in 58 young adult cancer survivors (median 20.5 years, range 18–29) and 27 comparators (median 20 years, range 17–29). The majority of survivors were female (62%), treated for a hematologic malignancy (61%), and a median of 5.5 years post‐completion of therapy (Table 1). The majority of comparators were female (74%). At the time of assessment, no survivors had evidence of active cancer or were receiving ongoing cancer‐directed therapies.

**TABLE 1:** Demographic, cancer, and treatment characteristics in young adult cancer survivors stratified by myopenia and frailty status.

Cohort characteristic	All survivors (*n* = 58)	Myopenic^b^ (*n* = 23)	Nonmyopenic (*n* = 34)	(Pre)Frail^c^ (*n* = 23)	Nonfrail (*n* = 31)
Median age at evaluation, year (range)	20.5 (18–29)	20 (18–29)	20.5 (18–29)	21 (18–29)	21 (18–29)
Female	35 (60%)	16 (70%)	18 (53%)	18 (78%)	14 (45%)
Cancer type					
Acute leukemia	24 (41%)	9 (39%)	14 (41%)	9 (39%)	12 (39%)
Lymphoma	11 (19%)	4 (17%)	7 (21%)	3 (13%)	7 (23%)
Non‐CNS solid	18 (31%)	6 (26%)	12 (35%)	8 (35%)	10 (32%)
Wilms' tumor	3 (5%)	0 (0%)	3 (9%)	0 (0%)	3 (10%)
Bone sarcoma	5 (9%)	2 (8%)	3 (9%)	3 (13%)	2 (6%)
Non‐rb soft tissue sarcoma	4 (7%)	2 (8%)	2 (6%)	1 (4%)	3 (10%)
Rhabdomyosarcoma	3 (5%)	2 (8%)	1 (3%)	2 (8%)	1 (3%)
Germ cell tumor	2 (3%)	0 (0%)	2 (6%)	1 (4%)	1 (3%)
Neuroblastoma	1 (2%)	1 (4%)	0 (0%)	1 (4%)	0 (0%)
CNS	5 (9%)	4 (17%)	1 (3%)	3 (13%)	2 (7%)
Treatment intensity					
ITR‐3—levels 1/2	14 (24%)	2 (9%)	12 (35%)	2 (9%)	10 (32%)
ITR‐3—level 3	37 (64%)	17 (74%)	19 (56%)	16 (70%)	19 (61%)
ITR‐3—level 4	7 (12%)	4 (17%)	3 (9%)	5 (22%)	2 (7%)
Total anthracycline dose (median mg/m^2^)^a^	125 (*n* = 55)	125 (*n* = 23)	150 (*n* = 31)	125 (*n* = 23)	150 (*n* = 29)
Comorbidities (≥1)	22 (38%)	11 (48%)	10 (29%)	11 (48%)	9 (29%)
Median time from treatment end, year (range)	5.72 (0.23–25.16)	4.32 (0.47–25.16)	6.83 (0.23–21.5)	4.32 (0.32–25.16)	6.57 (0.23–21.5)

Abbreviations: CNS, central nervous system; ITR‐3, Intensity of Treatment Rating Scale, version 3; (Pre)Frail, prefrail of frail survivors.

^a^ Calculated for 48 survivors who received anthracycline (doxorubicin equivalents).

^b^ One patient unable to be classified for myopenia given bilateral hip replacement and unable to obtain impedance data.

^c^ Four patients were unable to be classified for frailty due to incomplete patient‐reported outcomes for frailty assessments.

**FIGURE 1:**
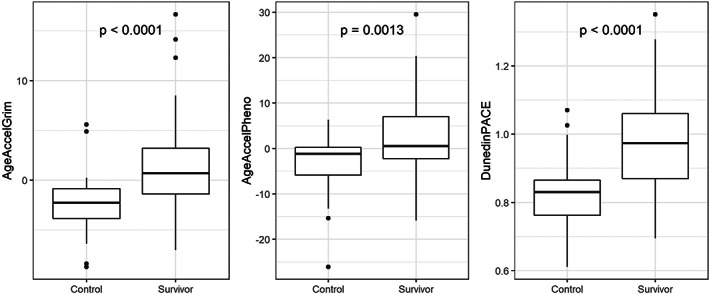
Comparison of biological aging measures between survivors (*n* = 58) and comparators (*n* = 27). Both age acceleration (AgeAccelGrim and AgeAccelPheno) and pace of aging (DunedinPACE) are higher among survivors versus comparators. Increased epigenetic age compared to chronological age is represented by AgeAccelGrim or AgeAccelPheno values >0 and DunedinPACE >1. *p*‐Values calculated using *t*‐tests.

Epigenetic age was significantly accelerated (measured epigenetic age > chronological age) among survivors versus noncancer comparators. On average, the epigenetic age for survivors was older than chronological age (denoted by a positive age accel value) while the average epigenetic age among comparators was younger than their chronological age (denoted by a negative age accel value) (1.5 vs. −2.4, *p* < 0.0001 [AgeAccelGrim] and 2.3 vs. −3.8, *p* = 0.0013 [AgeAccelPheno]). Pace of aging, a measure of decline in organ system integrity over time derived using Dunedin PACE, was also higher among survivors versus comparators (0.99 vs. 0.83, *p* < 0.0001) (Figure 1), which indicates 19% higher pace of aging for survivors.

**FIGURE 2:**
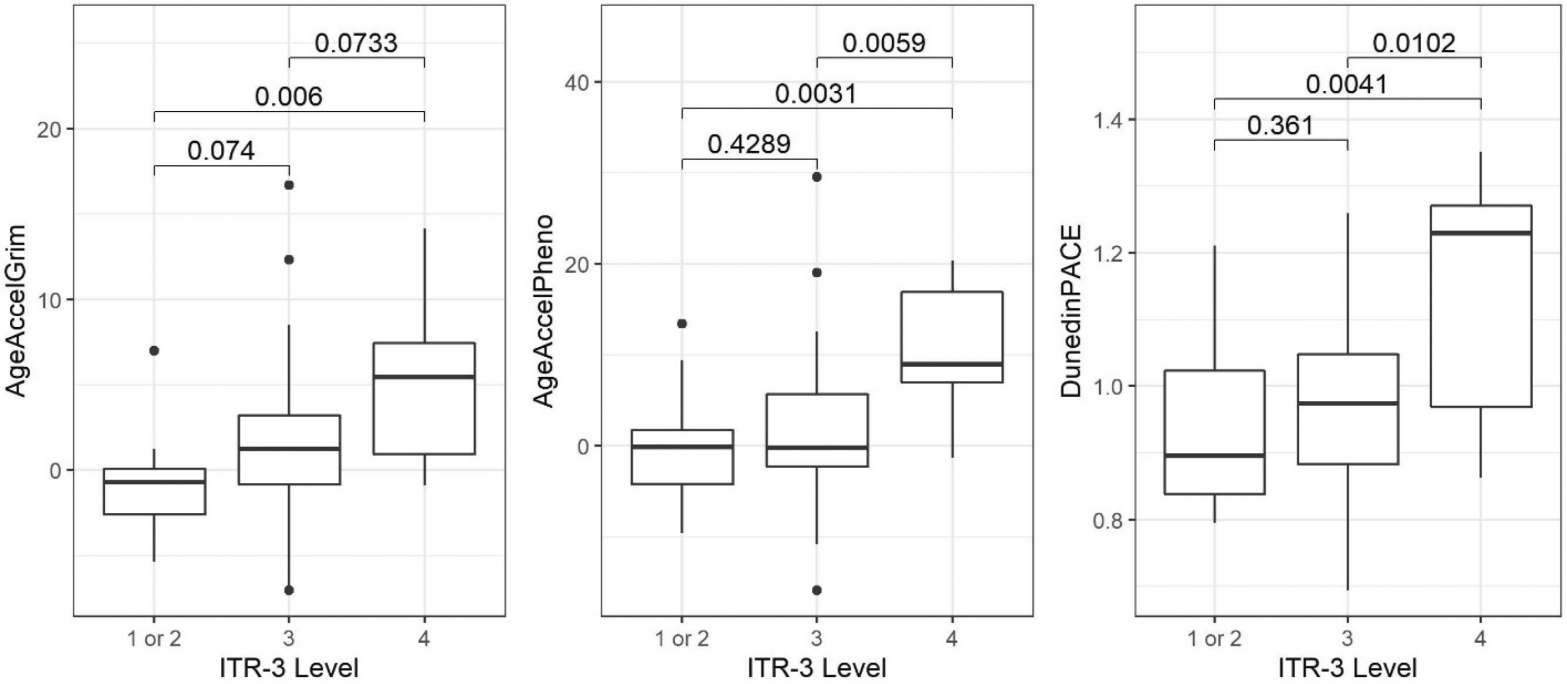
Biological aging measures by treatment intensity level (ITR‐3). Epigenetic age acceleration (AgeAccelGrim and AgeAccelPheno) and pace of aging (DunedinPACE) are increased among survivors treated with higher intensity therapy. *p*‐Values calculated using linear regression comparing treatment intensity level combinations.

In subgroup analyses of survivors, sociodemographic and treatment‐related factors were significantly associated with accelerated epigenetic age. Females had greater increase in PhenoAge compared to males (4.4 years vs. −0.7 years, *p* = 0.02) and experienced a 10% higher pace of aging compared to males (*p* = 0.009) (Table 2), though for females alone, the increased pace of aging did not reach statistical significance (1.03 [0.98, 1.08]) (Table 2). Participants who identified as either black or other race were epigenetically older and had a faster pace of aging compared to white participants (Table 2). While AgeAccelGrim was significantly higher among individuals who identify as Black or Other race and AgeAccelPheno was higher among those identifying as other, their increased DunedinPACE did not reach statistical significance (Table 2). The epigenetic age and pace of aging were greater in the patients who received treatment with highest intensity of regimens (ITR‐3 level 4) compared to other patients (Figure 2).

**TABLE 2:** Biological aging measures by patient, cancer, and treatment characteristics.

	AgeAccelGrim		AgeAccelPheno		DunedinPACE	
	Mean	95% CI	Mean	95% CI	Mean	95% CI
Female (*n* = 35)	1.5	(0.02, 3.1)	4.4	(1.4, 7.3)	1.03	(0.98, 1.08)
Male (*n* = 23)	1.4	(−0.9, 3.7)	−0.7	(−3.7, 2.3)	0.92	(0.87, 0.98)
*p*‐Value	0.92		0.02		0.009	
Black (*n* = 9)	4.3	(2.1, 6.6)	3.3	(−5.5, 12.0)	1.02	(0.92, 1.12)
White (*n* = 37)	−0.2	(−1.6, 1.2)	1.1	(−1.4, 3.6)	0.95	(0.91, 1.00)
Other (*n* = 12)	4.6	(1.5, 7.7)	5.5	(0.6, 10.4)	1.07	(0.97, 1.18)
*p*‐Value	0.007		0.26		0.04	
ITRS level 1, 2 (*n* = 14)	−0.9	(−2.6, 0.8)	−0.2	(−3.9, 3.5)	0.94	(0.87, 1.01)
ITRS level 3 (*n* = 37)	1.7	(0.1, 3.3)	1.7	(−1.0, 4.4)	0.98	(0.93, 1.02)
ITRS level 4 (*n* = 7)	5.1	(0.2, 9.9)	10.8	(3.7, 17.9)	1.13	(0.95, 1.31)
*p*‐Value	0.02		0.009		0.01	
≤5 years off Rx (*n* = 23)	2.8	(0.7, 4.9)	4.8	(0.4, 9.1)	1.02	(0.95, 1.09)
6–10 years off Rx (*n* = 18)	2.8	(0.5, 5.2)	1.8	(−1.8, 5.4)	1.03	(0.97, 1.09)
>10 years off Rx (*n* = 17)	−1.7	(−3.2, −0.2)	−0.3	(−3.1, 2.5)	0.90	(0.84, 0.96)
*p*‐Value	0.003		0.15		0.01	
Leukemia (*n* = 24)	1.1	(−0.6, 2.8)	1.6	(−1.3, 4.6)	0.97	(0.91, 1.04)
Lymphoma (*n* = 11)	0.1	(−2.5, 2.6)	2.8	(−3.1, 8.7)	1.03	(0.93, 1.13)
CNS tumors (*n* = 5)	3.3	(−1.7, 8.4)	9.7	(−4.7, 24.2)	1.01	(0.85, 1.17)
Other solid tumors (*n* = 18)	2.3	(−0.7, 5.4)	1.0	(−3.0, 5.0)	0.97	(0.90, 1.04)
*p*‐Value	0.49		0.19		0.75	
No comorbidities (*n* = 36)	0.7	(−0.4, 1.9)	2.1	(−0.2, 4.5)	0.97	(0.93, 1.02)
≥1 comorbidities (*n* = 22)	2.7	(−0.02, 5.4)	2.7	(−1.8, 7.2)	1.01	(0.94, 1.08)
*p*‐Value	0.13		0.79		0.38	
Nonmyopenic (*n* = 34)	0.2	(−1.3, 1.7)	−0.7	(−2.7, 1.4)	0.93	(0.89, 0.97)
Myopenic^a^ (*n* = 23)	3.3	(1.2, 5.4)	6.8	(2.7, 10.9)	1.07	(1.00, 1.14)
*p*‐Value	0.02		<0.001		<0.001	
Nonfrail (*n* = 31)	1.1	(−0.7, 2.9)	0.2	(−2.0, 2.3)	0.94	(0.90, 0.98)
Prefrail (*n* = 15)	2.7	(0.1, 5.3)	4.7	(−1.2, 10.6)	1.05	(0.95, 1.14)
Frail (*n* = 8)	1.6	(−2.2, 5.4)	6.2	(−2.7, 15.1)	1.09	(0.93, 1.24)
*p*‐Value	0.59		0.08		0.01	

*Note*: All *p*‐values report significance testing for overall difference among subgroups and were calculated using linear regression analysis. For variables with >2 categories, overall ANOVA *p*‐values are reported.

Abbreviations: CNS, central nervous system; ITRS, intensity treatment rating scale, version 3; Rx, treatment.

^a^ Myopenia defined as ≥1 standard deviation below sex‐ and age‐specific muscle mass population standards.

Both older epigenetic age and faster pace of aging were associated with physiological aging. Compared to survivors with normal levels of muscle mass, myopenic survivors had increased aging: AgeAccelGrim 3.3 years (*p* = 0.02), AgeAccelPheno 6.8 years (*p* < 0.001), and a 7% faster DunedinPACE (*p* < 0.001) (Table 2). One patient was unable to be classified for myopenia given bilateral hip replacement and unable to obtain impedance data. Four patients were unable to be classified for frailty due to incomplete patient‐reported outcomes for frailty assessments. Compared to nonfrail survivors, prefrail and frail [hereafter referred to as (pre)frail] survivors also had a significantly faster pace of epigenetic aging. Compared to nonfrail survivors who overall had a decelerated PACE (0.94), prefrail survivors demonstrated accelerated aging of 1.05 (12% increase over nonfrail [0.11/0.94]) and frail survivors an accelerated pace of aging of 1.09 (16% increase over nonfrail [0.15/0.94]) (Table 2).

Survivors with accelerated epigenetic aging reported greater physical function impairments compared to survivors without epigenetic aging acceleration. Scores on the SF‐36 physical function subscale and the composite physical function measure were significantly lower (poorer physical function) among survivors with aging acceleration (AgeAccelGrim or AgeAccelPheno > 0 or PACE > 1; Table 3). Additionally, survivors with accelerated PhenoAge also reported significant impairments in global health, vitality, and social functioning, greater limitations due to physical health, and worse pain.

**TABLE 3:** Mean SF‐36 domain and composite scores among young adult cancer survivors stratified by biological aging status.

Clock	Acceleration status	*n*	PF	GH	IPF	V	SF	Pain	IEF	MH	PCS	MCS
SF‐36 US population mean (SD) ages 18–24 years			92 (18.3)	76 (18.2)	89 (26.8)	63 (19.8)	84 (20.6)	81 (21.3)	83 (31.1)	75 (31.1)	53 (7.6)	49 (10.2)
AgeAccelGrim	Not accelerated	24	92	70	93	59	81	86	82	72	54	47
	Accelerated	34	77^	61	74*	55	78	79	79	76	46±	50
AgeAccelPheno	Not accelerated	27	90	71	92	64	87	89	85	76	54	50
	Accelerated	31	77*	58*	72*	50^	72*	76*	76	73	46±	48
DunedinPACE	Decreased pace of aging	35	89	68	88	61	83	85	84	75	52	50
	Increased pace of aging	23	75*	59	73	51	73	77	74	73	46*	47

*Note*: Survivors classified as having accelerated aging if AgeAccel measure is > 0 (epigenetic age > chronological age) or if DunedinPACE > 1 (ratio of epigenetic aging in years for each chronological year of aging). Student *t*‐test comparing survivors with and without epigenetic age acceleration: *p < 0.05; ^*p* < 0.01; ±*p* < 0.001.

Abbreviations: GH, global health; IEF, perceived impairment by emotional function; IPF, perceived impairment by physical function; MCS, mental health composite score; MH, mental health; PCS, physical health composite score; PF, physical function; SF, social function; V, vitality.

**FIGURE 3:**
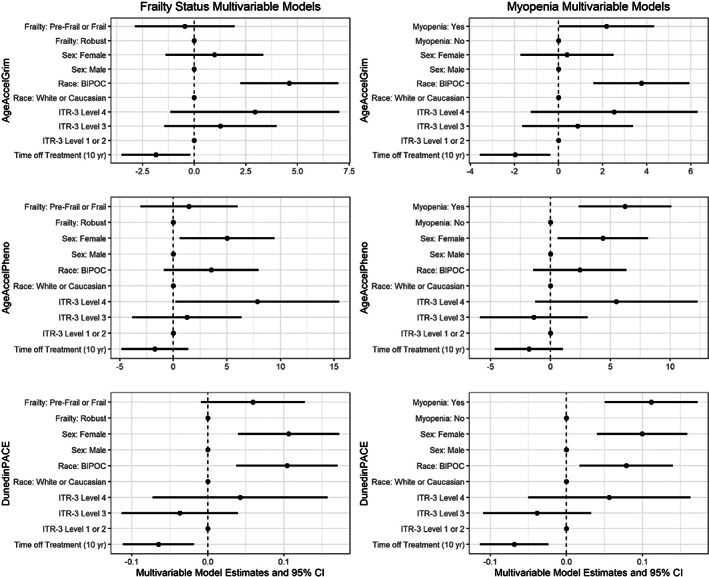
Multivariable linear regression models examining associations between biological aging measures and either frailty or myopenia in survivors. Time off treatment denotes change in biological aging measure for every 10 years post‐end of all cancer treatment. BIPOC, black, indigenous and people of color; ITR‐3, intensity of treatment rating, version 3.

In multivariable linear regression analysis adjusting for sex, race, treatment intensity, and time from end of treatment, age acceleration remained significantly higher among survivors with myopenia compared to survivors with normal levels of muscle mass (Figure 3). The increased age acceleration and pace of aging observed among (pre)frail survivors in bivariable analyses did not persist in multivariable analysis, although there was a trend toward faster DunedinPACE among (pre)frail survivors.

## 4 DISCUSSION

Among young adult cancer survivors, epigenetic age is significantly older (measured epigenetic age > chronological age) and pace of aging is significantly faster compared to similarly aged, noncancer comparators. Although very few comparators exhibited increased epigenetic age, the majority of survivors demonstrated an epigenetic age older than their chronological age and an accelerated pace of aging. For many survivors, epigenetic age was advanced greater than 5 years beyond their chronological age, with some by as many as 30 years (Figure 1). Using the DunedinPACE measure, the pace of biological aging among many survivors was advanced by as much as 25%–30% suggesting that for every 3–4 years of chronological aging, these survivors biologically age an additional year (Figure 1). These data clearly demonstrate young adult cancer survivors in our limited heterogenous population experience significant advancement in biological aging.

Epigenetic aging was observed using three DNA methylation‐based measures of biological age that have been derived using indicators of physiological aging and morbidity, suggesting that these clocks offer an easily assessable method for measuring premature biological aging in the cancer survivor population. Prior studies have reported epigenetic age acceleration in survivors of childhood cancers who were on average decades from completion of therapy.^38^, ^39^ Our study evaluated subjects early in survivorship care (median of 5.5 years post‐completion of treatment) demonstrating that changes in epigenetic aging are detectable early following treatment. As such, assessment of epigenetic age may become a useful tool during follow‐up to identify survivors with accelerated biological age and as a measure of efficacy for interventions to slow or reverse this process.

Increases in biological aging are hypothesized to precede physiological manifestations of aging such as loss of muscle mass or decreased physiological reserve.^19^, ^54^, ^55^ These processes may be accelerated among cancer survivors who receive chemotherapy and/or radiation and have been shown to develop physiological dysfunction, age‐related morbidities, and mortality much earlier in life compared to noncancer peers.^5^, ^6^, ^7^ Biomarkers of biological aging may provide a way to identify aging earlier among survivors. However, due to the study's design, it remains unclear if epigenetic aging precedes the sustained loss of muscle mass following treatment (and thus can serve as an early risk marker for this outcome) or if loss of muscle mass contributes to behavioral or functional changes that predispose to epigenetic aging. Further prospective studies are needed to understand aging and its associations with morbidity over time.

Inconsistent associations were observed between frailty status and epigenetic age. Epigenetic age was higher among (pre)frail versus nonfrail survivors; however, this association between frailty and epigenetic age did not persist after adjusting for intensity of treatment, sex, race, and time off therapy as was observed with myopenia. Indeed, myopenia may be the factor underlying the minor association between frailty status and epigenetic aging. The (pre)frail survivors who did not have myopenia met prefrailty criteria due to fatigue and low energy expenditure. It may be that epigenetic clocks are less sensitive to identifying survivors with these deficits; however, the clocks appear aptly suited to identify survivors with myopenia. When frailty develops in the setting of loss of skeletal muscle mass, it appears to be associated with greater epigenetic age.

Treatment‐related and sociodemographic factors were associated with greater accelerated aging among survivors. As expected, survivors with higher treatment intensity had older epigenetic age in the setting of heterogenous cancer types and prescribed treatment regimens. While not statistically significant across all ITR‐3 levels, likely due to the small sample size and limited power for this analysis, a graded increase in epigenetic age was noted with increasing intensity. As expected, myopenia and (pre)frailty were more common among patients treated with higher intensity. When controlling for treatment intensity, myopenia remained independently associated with greater epigenetic age acceleration and faster pace of aging (Figure 3) suggesting that physiological impairments, not treatment exposures alone, are associated with accelerated epigenetic aging and are important indicators of accelerated aging in young adult survivors. These findings are consistent with our hypothesis that because of the expected cellular damage associated with treatment, more intensive treatment regimens would lead to a clinical and epigenetic aging phenotype. After adjusting for intensity of treatment, epigenetic age remained older among females when compared to males and survivors who identify as black or another race when compared to white race. We acknowledge that there are factors aside from cancer treatment, for example, health behaviors and social drivers of health, that certainly also contribute to biological aging. Further studies with larger samples would allow for additional subgroup analyses and should consider analyses including social drivers of health.

The findings must be interpreted in the context of our study's limitations. Because of the observational design we cannot prospectively assess the time‐course of epigenetic changes and physiological impairment. As the study population was enrolled by a convenience sample of consecutive survivors presenting for survivorship care, these findings may lack broad generalizability to all young adult cancer survivor populations. Additionally, the study population included more survivors than comparators, which could affect our estimate for the difference in epigenetic age between survivors and comparators. This work was designed as a preliminary study to understand if epigenetic age is detectably higher than chronological age among survivors early post‐therapy, a finding confirmed by our results. Larger studies based on these initial findings are needed to better understand if these findings persist more broadly. Beyond the broader association with overall treatment intensity, the small sample size and heterogeneity in cancers and treatment types limits the ability to identify specific treatment modalities associated with epigenetic aging. The small sample size also limited our ability to examine differences in epigenetic aging among smaller subgroups by sociodemographic and cancer factors and limited the factors we were able to include in multivariable analysis. Finally, due to restrictions in the data available for comparators enrolled through the UNC platelet donation center, sex and age were the only characteristics available for description of the comparators.

In summary, our population of young adult survivors of childhood, adolescent and young adult cancers have a biological age significantly older than their chronological age as measured using DNA methylation‐based epigenetic clocks, which were derived using morbidity and mortality outcomes (AgeAccelGrim and AgeAccelPheno). Further, using a single time point measure for pace of aging (Dunedin PACE), many survivors experience a faster pace of aging. This is a first look at the epigenetic age in young adult cancer survivors recently off therapy. Survivors with adverse physiological changes such as myopenia or who report deficits in physical function have greater epigenetic age compared to survivors without these deficits. As these biological aging changes are evident early post‐therapy, their measurement may serve an early indicator of premature biological aging and may provide a way to measure the efficacy of interventions to slow or reverse this process.

## AUTHOR CONTRIBUTIONS

**Stephanie C. Gehle:** Conceptualization (supporting); formal analysis (lead); investigation (lead); methodology (supporting); writing – original draft (equal); writing – review and editing (equal). **Daniel Kleissler:** Data curation (lead); formal analysis (supporting); funding acquisition (supporting); methodology (supporting); project administration (supporting); writing – original draft (supporting); writing – review and editing (supporting). **Hillary Heiling:** Data curation (equal); formal analysis (equal); methodology (equal); validation (equal); writing – original draft (supporting); writing – review and editing (supporting). **Allison Deal:** Data curation (equal); formal analysis (equal); methodology (equal); validation (lead); writing – original draft (supporting); writing – review and editing (supporting). **Zongli Xu:** Conceptualization (supporting); data curation (supporting); investigation (equal); methodology (equal); resources (equal); writing – review and editing (supporting). **Vanessa L. Ayer Miller:** Data curation (supporting); project administration (equal); writing – review and editing (supporting). **Jack A. Taylor:** Conceptualization (supporting); data curation (equal); investigation (equal); methodology (equal); resources (lead); writing – review and editing (equal). **Andrew B. Smitherman:** Conceptualization (lead); data curation (lead); formal analysis (lead); funding acquisition (lead); investigation (lead); methodology (lead); project administration (lead); resources (lead); supervision (lead); writing – original draft (equal); writing – review and editing (equal).

## CONFLICT OF INTEREST STATEMENT

The authors have no conflicts of interest relevant to this article to disclose.

## ETHICAL APPROVAL

All study activities have been approved by both the UNC Lineberger Cancer Center Protocol Review Committee and the UNC Institutional Review Board and adhere to the US federal policy for the protection of human subjects.

## Data Availability

The data that support the findings of this study are available from the corresponding author upon reasonable request.
